# Chest Pain in Adolescent Japanese Male Mimicking Acute Coronary Syndrome

**DOI:** 10.1155/2014/176520

**Published:** 2014-08-18

**Authors:** Sachin K. Gupta, Zahra Naheed

**Affiliations:** ^1^Department of Pediatrics, John H. Stroger, Jr. Hospital of Cook County, Chicago, IL 60612, USA; ^2^Department of Pediatric Cardiology, John H. Stroger, Jr. Hospital of Cook County, Chicago, IL 60612, USA

## Abstract

Acute chest pain with very elevated troponin level and abnormal EKG in adult population is considered sine qua non to acute coronary syndrome (ACS) unless proved otherwise. Similar presentation in adolescent population is seen less often but raises suspicion for ACS. Most common etiology for chest pain with cardiac enzyme elevation in adolescent population is usually viral myopericarditis. The adolescent population presenting with chest pain and elevated cardiac enzymes should be carefully evaluated for ACS and other etiologies including myocarditis, myopericarditis, pulmonary embolism, acute rheumatic fever, and trauma. We report one Japanese adolescent male with mycoplasma pneumoniae myocarditis who presented to the ER with chest pain, elevated cardiac enzymes, and abnormal EKG.

## 1. Case

A 17-year-old Japanese origin male presented to the pediatric ER with sudden onset of sharp substernal chest pain. The pain started 24 hours prior to admission, described as nonradiating, sharp, constant pressure over the left chest with the intensity rating of 10 out of 10. There were no other associated symptoms like shortness of breath, cough, diaphoresis, nausea, or vomiting. There was history of one episode of loose stool and one fever spike of 101 F within the last 24 hours of admission. Acetaminophen was given at home without much improvement. No significant risk factors for coronary artery disease were identified. There was no family history of coronary artery disease or sudden cardiac death. Patient denied any substance use.

On examination, he was afebrile with heart rate of 74 bpm, respiratory rate of 18/min, BP of 135/50 mm hg, and oxygen saturation of 96−98 percent on room air. Cardiac examination revealed normal S1S2, no murmur, no rub, gallop, or distended jugular venous pulse. There was no chest wall tenderness or hepatomegaly.

**Figure 1:**
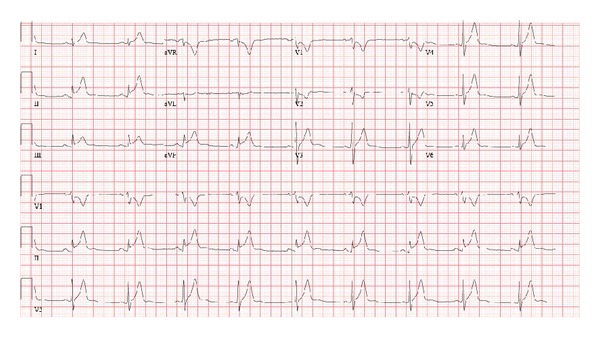
EKG showing sinus tachycardia, ST elevation in lead II, III, and aVF, and ST depression with T wave inversion in V1 and V2.

Stat EKG and cardiac enzymes were ordered. The initial EKG (Figure 1) showed sinus tachycardia, ST elevations in lead II, III, and aVF, and ST depression with T wave inversion in V1 and V2. Troponin I and CK-MB done on arrival were markedly elevated (troponin I 17.1 ng/mL, normal < 0.034). The toxicology screen was negative. A stat echocardiogram on arrival showed structurally normal heart with normal origin of coronary arteries, normal left ventricular ejection fraction without regional wall motion abnormalities, and no pericardial effusion. However, two echocardiograms that followed at 8–12-hour intervals showed dilatation of left atrium and left ventricle with trace mitral regurgitation. Further workup including CRP, ESR, nasopharyngeal and throat viral cultures, EBV DNA, enteroviral culture (rectal), and serum titers (coxsackie, adenovirus, echovirus, mycoplasma, and influenza A and B) was sent. Cardiac MRI (Figure 3) was performed and showed biventricular hypokinesis with extensive multiple foci of curvilinear and oval shaped contrast hyperenhancement involving the central portions of the anterior, lateral, and inferior wall of LV and posterior septal and anterior wall of RV with relative sparing of the endocardial portions of the myocardial walls most suggestive of myo/myopericarditis.

**Figure 2:**
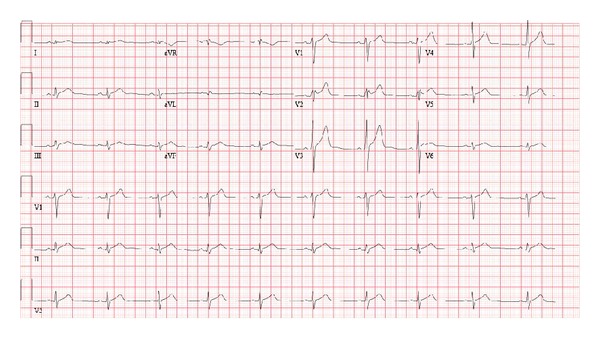
EKG showing marked improvement in ST elevation, low voltage QRS, and rightward axis.

**Figure 3:**
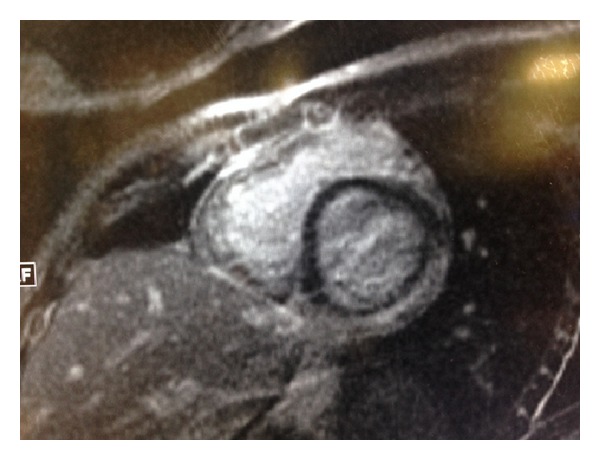
Cardiac MRI showing multiple foci of curvilinear and oval shaped contrast hyperenhancement involving the central portions of the anterior, lateral, and inferior wall of LV with relative sparing of the endocardial portions of the myocardial walls.

Initial chest pain subsided within few hours after admission after giving morphine and sublingual nitroglycerine. The patient continued to have milder grade, intermittent chest pain, and low fever which improved over the following 3-4 days of admission. Patient was given course of IVIG in view of uncertain diagnosis and suspicion of Kawasaki disease (as patient was having persistent fever and of Japanese ethnicity). Mycoplasma IgM titers were high (1193 *μ*/mL, normal < 770). Patient was subsequently treated with course of azithromycin. Troponin I gradually showed significant downward trend (0.25 ng/mL on day 6 of admission). The EKG on second and third days (Figure 2) showed gradual improvement in ST elevation with low QRS voltages, rightward axis, and subsequent normalization of ST elevation in later EKG's. The patient remained asymptomatic after discharge for 10 weeks, till the time of acceptance of this case report.

## 2. Discussion

Considering the patient's young age, no risk factors for coronary artery disease and other cardiac diseases and no history of coagulation disorders or substance abuse and trauma coronary artery disease were less likely. With the elevated serum titers of mycoplasma and cardiac MRI findings of myocarditis, diagnosis of Mycoplasma pneumoniae myocarditis was made.

The cardiac origin chest pain represents a very small proportion (1.5–3 percent) of all pediatric visits to ER for chest pain [1–3]. Most common cardiac etiologies in healthy adolescent population includes myopericarditis especially viral myopericarditis in summer months and less often bacterial myopericarditis and even less often myocarditis without apparent pericardial involvement. The cardiac enzymes elevation can also be seen in cases of myo/myopericarditis [4–6].

Mycoplasma pneumoniae myo/myopericarditis has been reported in adolescent population [7–17]. There have been case reports of mycoplasma pneumoniae myo/myopericarditis without associated respiratory symptoms [17]. Mycoplasma pneumoniae is a common cause of community acquired pneumonia in young adults. The presence of exanthems and gastrointestinal symptoms is also quite common, but mycoplasma-associated carditis (myo- or pericarditis) is an uncommon complication especially in pediatric population.

These patients can be effectively treated with macrolides (azithromycin). Tetracyclines and fluoroquinolones may be considered in older children and adolescents. In our case, patient was treated with azithromycin course, after diagnosis was made.

Long-term complications have been reported with mycoplasma myocarditis. A review by Pönk [7] in 1979 reported 43% of the patients with cardiac sequelae. The relatively recent review by Paz and Potasman described a complication rate of less than 30% and attributed it to better antibiotic coverage [8].

Our case represents uncommon presentation of cardiac origin chest pain in adolescent population. Although it is reasonable to consider ACS as first diagnosis in adult patients and adolescents with risk factors, in other adolescent patients especially with recent history of febrile illness, infectious etiology should be considered, as was diagnosed in our patient. Serial electrocardiogram and cardiac enzymes are useful diagnostic aid during initial period. The detailed workup for infectious etiology is important for directed treatment and future prognosis. Cardiac MRI has emerged as reliable noninvasive diagnostic study to further support the diagnosis and if available, should be performed after stabilization of patient.
